# Observations on the Puerperal Fevers Which Occurred at the Maternité of Paris in 1829; on the Various Modes of Treatment Employed, and Particularly on Mercurials, Emetics, and Bloodletting

**Published:** 1830-07

**Authors:** M. Tonnellé


### PUERPERAL FEVERS.

*Observations on the Puerperal Fevers which occurred at the Maternité of Paris in* 1829*; on the various Modes of Treatment employed, and particularly on Mercurials, Emetics, and Bloodletting.* By M. Tonnellé.

M. Tonnellé has had so much experience upon the very important subject of puerperal fevers, and he delivers his opinions with so entire a freedom from partiality to any preconceived opinion of the pathology of the disease, and of the treatment it requires, that we are sure we shall confer a benefit upon our readers by giving them an abstract from the *Archives** of the excellent essays he has published. It will be seen that M. T. corroborates the opinions which have been lately published by Dr. Lee and other English practitioners, who have endeavoured to arrive at correct notions of the pathology of puerperal fever.

The points to which our attention is directed are, 1st, the organic alterations which are detected in puerperal fever; 2d, a history of the symptoms; 3d, a description of the treatment. Before these subjects are discussed, some general reflections are made upon the causes of the disease.

The puerperal fevers observed at the Maternité in 1829 were more frequent and severe than had ever been known since the foundation of the institution. The disease frequently occurred in an epidemic form, particularly in January, May, August, September, and October; and during these months it raged with great violence. It might, at first, be supposed that the developement of the disease depended upon the cold and moist weather which reigned

* Mars 1830.

throughout the year; but, as a proof that the disease did not entirely depend upon these causes, it is stated that, during the keen dry coldness of January, puerperal fevers were very frequent; but, on the other hand, in December, when the weather appeared to be precisely the same, but very few cases occurred. The influence of moist weather is equally doubtful: for, although these fevers happened frequently during the summer, which was cold and rainy, they were very rare at certain intervals, during which the conditions of the atmosphere remained the same, and very common during the long and remarkable dryness of the spring. The vitiated state of the air in hospitals, and those moral affections which are usually considered to play so important a part in the production of puerperal fevers, cannot fairly be admitted as causes: they act in a uniform and constant manner. The disease, on the contrary, usually occurred very irregularly, raging during a week or a month, then suddenly disappearing, and again becoming frequent. Much has been said by authors of the influence of peculiarity of constitution, previous diseases, and the duration and difficulties of labour; but it must be evident that either of these causes can only refer to individual cases of the disease, and that they cannot in any manner explain the simultaneous occurrence of a great number of cases.* There was no reason whatever to believe that the disease spread by contagion. It often happened that patients were attacked with puerperal fever directly after delivery, although they were in insulated wards, and had no communication with the other women. M. Tonnellé would, therefore, refer the origin of the disease to some more general and moveable cause, to some secret influence of the atmosphere, which, although impenetrable to our senses, is no less certain.

*Of the morbid appearances.* Inflammation of the peritoneum was the most frequent alteration that was observed after puerperal fevers; but it is erroneous to suppose that it always exists. Sometimes this membrane was of a perfectly natural appearance, and the most minute investigation failed to detect any appreciable marks of disease; and it was particularly in the most severe and rapidly fatal cases that this integrity of the peritoneum was remarked. In these instances there mostly existed some alteration of the womb, or of its vessels, or the parts connected with it.

* It was not uncommon to see ten or twelve women attacked during a day or night with puerperal fever.

The anatomical character of the peritoneal inflammation was not always the same. Sometimes no fluid was effused into the abdominal cavity, but upon the surface of the intestines there was a deep blush of red colour, and a delicate white membrane formed, producing adhesion between the different parts. Most frequently a certain quantity of fluid was found in the abdomen, mingled with shreds of albumen and false membrane, of a yellowish colour. In some instances pure pus was seen. The inflammation of the peritoneum was mostly confined to the hypogastric region, and around the uterus; and when it was more extended, it was still more intense in these parts. In a few rare examples, particular parts of the peritoneal membrane were affected, as upon the surface of the liver, the mesentery, and omentum. When the last part was inflamed, it was thickened and hardened so much that it could be felt during life through the parietes of the abdomen. The false membranes that were found were sometimes of a brown colour, which for a long time was supposed to indicate a gangrenous state. The recent improvements in pathological anatomy have shown this opinion to be erroneous, and it would have been unnecessary to mention the circumstance, if the same mistake had not lately been fallen into by some writers on peritonitis.

The *morbid appearances of the uterus* may be classed under three principal heads: 1st, simple inflammation of the uterus, and the parts connected with it; 2d, suppuration of the veins and lymphatics; 3d, a softened or putrescent state. The internal surface of the uterus was generally covered with a putrescent secretion, of a reddish brown colour, the fetor of which was often almost insupportable. There were also frequently found upon the internal surface of the uterus a number of small greyish granulations, presenting an aphthous appearance. In other cases there was a layer of thick yellow pus lining the whole or a part of the uterus. Upon the peritoneal covering of the uterus were often seen small irregular vesicles, formed by the effusion of a sero-purulent fluid, or true pus, beneath the membrane. When these vesicles were ruptured, the surface of the uterus was partly deprived of its external tunic, as the skin is of the epidermis after the application of a blister. An altered condition of the proper tissue of the uterus was rare, with the exception of a softened or putrescent state. But if, in dissection, the parts were not very carefully examined, the collections of matter, which really existed in the veins or lymphatics, were easily mistaken for small abscesses in the substance of the uterus itself. In the substance of the broad ligaments of the uterus pus was often found, and also in the fallopian tubes. The ovaria were either injected with blood or contained serum, accompanied by tumefaction and a softened state of structure. Sometimes there was a general purulent infiltration of these organs, with great enlargement and remarkable friability of their tissue. In most cases the pus was disseminated throughout the ovaria, but in a few instances it was confined to one part. In a woman who died at an advanced period of the disease, an abscess of the ovarium communicated with the rectum by ulceration. In another instance a similar abscess had ruptured in the cavity of the peritoneum. The ovaria often contract adhesions with the abdominal parietes, and the pus they may contain is then spontaneously discharged externally. Ruisch, Delamotte, and Desormeaux have related such cases.

*Of suppuration of the veins and lymphatics of the uterus.* M. Dance first pointed out inflammation of the uterine veins, and the serious consequences which hence arise in cases of puerperal fever, in a memoir in the Archives générales: but M. D. had only the opportunity of collecting a few scattered cases. M. Tonnellé is not aware that suppuration of the lymphatics has been either observed or described before. He considers this morbid state as important as that of phlebitic inflammation, since the symptoms and serious consequences of both are similar. Suppuration of the veins and lymphatics of the uterus is so frequent, that it was met with in nearly three out of five cases of puerperal fever which occurred. It is, in fact, almost as constant as peritonitis. Sometimes it existed alone, but more frequently it was combined with some of the preceding morbid alterations. In most instances the vessels of the uterus were alone affected, but occasionally the adjacent veins of the thoracic duct were implicated. In some examples pus was contained in a few vessels only, but in most all the vessels were filled, so that no portion of the uterus could be divided without seeing the fluid pour out in the form of numerous small drops from the orifices of the vessels. It was principally on the lateral parts of the uterus, and near the origin of the broad ligaments, where there are numerous veins and lymphatics, that this morbid state was detected. It was rare near the insertion of the placenta, where M. Dance, however, appears most frequently to have observed it. The lymphatic vessels, in a state of suppuration, are easily distinguished from the veins by their superficial position on the sides of the uterus, and upon the surface of the broad ligaments; by the tenuity of their coats, and by the whitish, milky appearance which they give to the membrane covering them; and also by their proximity to the large veins, their tortuosities, and remarkable enlargements which they present at various distances. These enlarged portions of the lymphatics sometimes formed small pouches, filled with cream-like pus, and large enough to admit a cherry-stone, or even a kidney-bean. Great attention is required to distinguish these appearances of the lymphatics from abscesses of the proper substance of the uterus. The internal membrane of the lymphatics was occasionally thickened and irregular, but it usually preserved the natural polish, and was altered only in colour, which was of a yellowish hue. M. Tonnellé is not, however, prepared to admit that the pus is not secreted by the internal membrane of the lymphatics; for their pellucid membranes present very few appreciable alterations in the best-marked states of inflammation. In ordinary phlebitis, the morbid appearances are found almost exclusively upon the external membrane, which is of a cellular structure, and which becomes so swollen and thickened as to give to the veins the appearance of arteries. When this membrane does not exist, or is not perfectly formed, as in the parenchymatous substance of organs and in the venous sinuses of the cranium, inflammation leaves less well-marked traces. There is no reason why the lymphatics should not be as susceptible of inflammation as the veins. We find in the former vessels all the conditions which appear to favor the developement of inflammatory action in the latter: a great augmentation of their capacity; an increase of their vital power; various contusions during the act of labour; an immediate contact with the decomposed matter which covers the internal surface of the uterus; absorption of puriform, acrid, and fetid secretions, &c.

Suppuration* of the veins and lymphatics of the uterus were usually accompanied by a certain number of symptoms, which, as M. Dance has well observed, are peculiar to that kind of affection. The presence of pus in these

* M. Tonnellé does not wish that the term "suppuration," as here applied, should be taken as implying more than the simple fact of pus being contained in the vessels, without prejudging the origin of it. The practical facts which he subsequently relates will, he thinks, settle the question of where the matter is formed.

vessels, and its consequent transmission into the general circulation, rapidly produced an evident and palpable infection of the whole mass of blood; and hence arose a certain group of the most serious symptoms, which gave to the puerperal fever a special and characteristic form. The following cases, taken from many others, are adduced to prove this fact.

Case I. Victor Ano, æt. twenty-two, of a good constitution, had a favorable delivery at the Maternité, in the beginning of July. Four days after her labour, she complained of shivering and pains in the hypogastrium, which were immediately opposed by the application of fifty leeches. Next day, the belly was swollen and extremely tender; face flushed; pulse hard, quick, and full; lochia suppressed; no milk. Leeches were again applied, and an oily mixture and a hip bath ordered. The third day from the attack, a decided change occurred: Profound prostration; face pale and drawn; eyes half closed; tongue dry; pulse small, quick, and irregular; the patient articulated with difficulty; occasional shiverings; involuntary discharge of feces. In the evening, delirium. Fourth day, the body was covered with a clammy sweat; extremities cold; and the woman soon sank, and died.

Mercurial frictions with three ounces of the ointment each day, and calomel with hyoscyamus, were prescribed by M. Desormeaux during the latter period of the disease.

*Dissection, twenty-four hours post mortem.* Internal surface of the uterus covered with putrid and fetid matter, but it did not appear to be altered in structure. Upon dividing the body of the uterus, the open orifices of several sinuses were seen, full of yellow and well-formed pus, which flowed out abundantly from all parts upon the slightest pressure. These sinuses opened both on the right and left side, towards the origin of the ligamenta lata, where a numerous collection of veins and lymphatics were seen filled with the same fluid; but at this point the alteration abruptly terminated. The hypogastric and ovarian veins were full of brown fluid blood, but in them there was no vestige of pus. At the inferior part of the peritoneal cavity there were a little sero-purulent fluid, and some false membranes. The other organs were healthy.

In this case, as in all the other similar examples, two distinct orders of symptoms occurred: the one peculiar to inflammation of the veins of the uterus, or of the uterus itself; the other, on the contrary, of a very different nature, and indicating an absorption of pus and infection of the general constitution. The practical utility of this distinction must be obvious: for, if antiphlogistic means should be employed with energy during the first period, the second requires a very different plan of treatment. If we study the cases of *putrid* puerperal fever left us by ancient writers, we should find in them the most striking analogy with the symptoms of internal phlebitis, as observed in the present day, so that we cannot doubt that the disease formerly described owed its character of *putridity* to the absorption of pus, as is now found to be the case. "In putrid puerperal fevers," says Leake, "the belly rapidly swells, the countenance of the patient alters, the tongue is dry, hands tremble, lips livid, nostrils dilated, the cheeks are often of a deep red colour, the patients sink into a state of prostration, the pulse becomes excessively rapid, and lastly tremulous." These are the characters which we daily witness as the accompaniments of the second stage of uterine phlebitis. Leake, indeed, who did not admit the idea of a metastasis of milk, which was then generally adopted, suspected the real origin of the symptoms. In his opinion, puerperal fever was primarily the result of inflammation, and if in the latter stages it assumed a putrid form, it was from the absorption of various purulent fluids secreted in the abdomen, which fluids, by mingling with the general mass of blood, excited a putrid fermentation. Whether these ideas arose from practical observation or hypothetical speculation, may be doubted; but they are remarkable for the period in which they were promulgated. If we compare the preceding case with others observed by Dance, we perceive that phlebitis runs a very rapid course; but in some instances the disease is still more quickly formed, and appears almost instantaneously, and at once destroys life.

CASE II. *Puerperal Fever, with Uterine Phlebitis, rapidly fatal.*

Marie Cons, æt. twenty-eight, of a good constitution, was delivered, after a favorable labour, at the Maternité, on the evening of December 26th.

27th.—Well in the morning. Evening: some wandering pains in the abdomen.

28th.—Pains more severe in the morning. Lochia suppressed entirely; they had previously been abundant. Face pale and anxious; tongue dry; pulse small, hard, and quick.—Fifty leeches were applied to the hypogastrium; hip bath. Blood flowed freely from the leech bites, but the pains were not relieved. During the day, the patient was extremely anxious and agitated. In the evening she became delirious, and fell into a profound stupor. The skin was soon covered with clammy perspiration; extremities became cold, and death happened about twenty hours from the invasion of the disease.

*Dissection.* Uterus not contracted, and filling nearly the whole of the pelvis; its internal surface covered with a brown and very fetid matter. The cervix uteri, ligamenta lata, and subperitoneal cellular tissue, were infiltrated with a large quantity of well-constituted pus. Most of the veins and lymphatics were filled with the same fluid, and it flowed from them in a multitude of large drops, wherever an incision was made. Fallopian tubes swollen and very red. Ovaria softened. Lymphatic glands of the pelvis and loins much enlarged. Peritoneum and other parts healthy.

The enormous quantity of pus contained in the uterine vessels, and the inevitable absorption of it, satisfactorily accounted for the severity of the symptoms, and the rapid destruction of the patient. But how can we explain the singular rapidity with which the pus must have been formed? In the most acute external inflammations, suppuration is not established for many days. In the above case, on the contrary, hardly twenty hours elapsed from the invasion of the disease, and already was the uterus filled with pus. This terrible rapidity in the march of puerperal fever is rarely observed in sporadic affections. It is seen, almost exclusively, in the course of various epidemics, and we must therefore seek for the cause, not in idiosyncrasy, or individual predisposition, but in the character of the epidemic which stamps upon the disease a peculiar form, modifies the morbid alteration of parts, renders a different plan of treatment necessary, hastens or procrastinates the duration of the disease, and augments or diminishes its danger, according to certain conditions which we are unable perfectly to explain. Accustomed as we are at the present time to regard diseases as insulated and unconnected occurrences, and scarcely ever in their mutual connexion, and but little in the habit of observing epidemic maladies, which the progress of civilization happily renders every day less frequent, we are unwilling to admit the influence of general causes which are not evident to the senses: but, unless we do so, how can we ever comprehend epidemic diseases? In a particular year, or month, the progress of puerperal fevers is marked by wonderful rapidity. At another period the same disease runs a slow and progressive course. At one time the symptoms are mild, at another dreadfully severe.

In one epidemic, particular morbid alterations are observed; in another, certain plans of treatment are unusually successful. How can we explain these various general facts, without admitting the existence of a general cause which predominates over individual peculiarities? M. Tonnellé allows that these ideas are not yet sufficiently supported; but he hopes, in the course of his essays, to develope them satisfactorily, and to prove that they are well founded.

CASE III. *Puerperal Fever, with Pus in the Lymphatics of the Uterus and in the Thoracic Duct. Considerable Tumefaction and Softening of the Lymphatic Glands of the Groin and Lumbar Region.*

— Lor, æt. thirty-one, of good constitution, entered the Maternité the 1st of July, 1829, and was delivered of her first child the 25th of August. On the day of her confinement, she had repeated shiverings, pain in the hypogastric and lumbar regions, and intense fever. M. Desormeaux ordered her to be bled copiously, and fifty leeches to be applied to the abdomen.

The second day, the pains were excessive, lochia suppressed, nausea, redness of the face, and fever.—Venesection and leeches repeated.

Third day, countenance anxious, alternate agitation and depression, delirium, tympanitic state of the belly, incontinence of urine and feces, small and irregular pulse. Died in the evening.

*Dissection.* A quantity of pus was infiltrated between the laminæ of the ligamenta lata of the uterus. The lymphatics, which were filled with the same liquid, formed upon the edges of the broad ligaments, and upon the sides of the uterus, large, white, tortuous, superficial cords, with very thin coverings. These vessels were swollen at different points, and surrounded the large venous trunks, which were entirely empty. The glands in the groin and lumbar region were as large as pigeons' eggs, and on the anterior part of the spinal column they formed voluminous masses; their tissue had a greyish appearance, and it was infiltrated with pus, and easily broke under the finger. The thoracic duct, of the size of a swanquill, was filled with thick yellow fluid, resembling pus. In the cavity of the abdomen was about half a pint of floculent serum. All the other parts were healthy.

CASE IV. *Suppuration of the Lymphatics of the Uterus and of the Thoracic Duct.*

Sophie G., æt. twenty-one, of good constitution, was admitted October 28th, 1829, in the eighth month of her pregnancy. The inferior extremities were œdematous.

Nov. 7th.—Shivering, vomiting, and headach.

8th.—Patient was attacked with convulsions, which continued in frequent paroxysms for two days. She then became profoundly comatose. These symptoms were subdued by vigorous treatment. Strong labour pains came on, and she was delivered of a living child, without any untoward occurrence. Until the next day, she went on well: she was then attacked with pains in the hypogastrium, and had some shivering. Fifty leeches were applied to the abdomen.

Third day, the lochial discharge ceased; violent pains in the abdomen; vomiting; high fever. Leeches repeated.

Fourth day, delirium; constant state of agitation; impeded articulation. These symptoms were followed by great depression, and in the evening the patient sunk, and died.

Mercurial frictions in large doses, and blisters to the legs, were the means principally employed during the last two days.

*Dissection.* Internal surface of the uterus of a brown colour, and superficially softened. Cellular tissue connecting the peritoneum to the body of the uterus, and the ligamenta lata, were infiltrated with pus. Most of the lymphatics were filled with the same fluid, and formed, as in the preceding case, large knotty superficial trunks. This appearance was not confined to the lymphatics of the uterus, but extended to most of those vessels in the abdomen which were swollen and of a milky colour. The thoracic duct was greatly dilated, and filled with pus. There was a large quantity of sero-purulent fluid in the abdomen. Left ventricle of the heart slightly hypertrophied. Other parts healthy.

Many other cases similar to these were seen, but they were so analogous as to render a detail of them unnecessary. Was the pus formed primarily in the vessels, or was it carried into them by absorption? Both opinions may be supported; for, on the one hand, pus was found in the cellular tissue, and particularly in the substance of the ligamenta lata, and therefore the lymphatics might have obtained from these parts the matter which they contained.

But, on the other hand, there was no kind of proportion between the liquid infiltrated in the uterus and that which filled the blood-vessels; besides which, the lymphatic glands presented various marks of inflammatory action.* The question, however, is not so important as might be imagined. The danger arises from the presence of a certain quantity of pus in the vessels, from whence it is transported to various other parts; and whether the pus is carried into the vessels by absorption, or is formed in them spontaneously, the effects are precisely the same. In the previous cases, the phlebitic inflammation was confined to the uterus; but it frequently extends beyond that organ, and affects the adjacent veins. Sometimes it attacks the latter alone, the uterine veins remaining healthy to all appearance; which is shown by

CASE V. *Puerperal Fever, with Inflammation of the Hypogastric, Crural, and Iliac Veins.*

Marie Mart..., æt. twenty-eight, of healthy constitution, was attacked, on the third day after a favorable labour, with symptoms of severe puerperal fever. By very abundant local bleeding, the disease was quickly checked, and appeared to yield entirely on the eighth day; but there soon followed headach, singing in the ears, great agitation and depression, and occasionally delirium.

On the thirteenth day, the patient had shiverings and pains in the abdomen, which had for some time entirely disappeared. Two ounces of mercurial ointment were rubbed in each day. She was again relieved, and appeared nearly convalescent. She had some appetite; but still each day there was a slight febrile attack, and she continued getting thinner.

22d.—Inferior extremities very œdematous.

29th.—Pains in the abdomen, vomiting, and high fever.

31st.—Extreme prostration. Death.

*Dissection.* Peritoneum lined with false membrane, by which the intestines adhered firmly together. Abdominal cavity filled with pus. Uterus perfectly contracted, and of healthy appearance. The hypogastric veins much dilated,

* In the cases published by Dr. LEE, in the Med.-Chir. Transactions, the appearances of inflammation in the veins were too strongly marked to admit of doubt. Dr. Lee recently assisted us in the post-mortem examination of a patient who died of puerperal fever, and whose symptoms very closely resembled those described by M. Tonnellé. The uterine and spermatic veins were filled with pus, but there were no appreciable signs of inflammation in the structure of the vessels.—EDITOR.

and filled with a quantity of thick greyish pus. The crural veins, the iliac, and a part of the inferior cava, contained a dense coagulum, in the centre of which was a certain quantity of pus. The circulation must here have been completely obstructed. The parietes of all these vessels were much thickened, unequal, and rough. The upper part of the inferior cava, which was unaltered in its appearance, was quite empty, white, and remarkably contracted. The other organs were natural.

In this case we see phlebitis occupying the large venous trunks adjoining the uterus, without any trace of disease in this organ itself. It must, therefore, be admitted that inflammatory action may originate in those vessels, unless we suppose that it had terminated by resolution in the uterine veins, and continued in the other. This would be entirely a gratuitous hypothesis, and would not admit of defence. Phlebitis appeared to develope itself from the commencement of this long disease. The general symptoms which arose on the ninth day, as the singing in the ears, extreme agitation, prostration, and delirium, strongly indicated it; but, notwithstanding the extent of the inflamed surfaces, these symptoms of purulent infection were not so severe as in the former instances. At a first view, this fact may seem inexplicable; but, if we reflect upon the case, the reason will be obvious. A certain quantity of pus was first carried into the torrent of the circulation, but soon the coagulum, which formed in the crural, iliac, and cava veins, by obstructing the circulation, circumscribed and as it were imprisoned the pus, and consequently opposed the production of those severe symptoms which would otherwise have arisen from absorption. The œdematous swelling of the inferior extremities, the adherence and solidity of the coagulum which filled the vessels in question, and, lastly, the emptiness and contracted state of the inferior cava, leave no doubt as to the circulation having been interrupted.

In the preceding cases, we have seen absorption of pus, the consequence of inflammation of the uterine vessels, produce the most severe symptoms, and destroy the patient in the midst of a general derangement of the functions, without any appreciable lesion of the tissue of the organs: but it often happens that the morbid condition of the fluids reacts upon the solids, and inflicts upon them very serious lesions; of which the following case is an example.

CASE VI. *Puerperal Fever, with Uterine Phlebitis; Perforation of the Stomach; Softening of most of the Organs.*

E. Porch..., æt. twenty-five, of healthy constitution, was delivered, after a good labour, at the Maternité, July 20th, 1829. On the third day from her confinement, she had shiverings and severe pains in the abdomen. Forty leeches applied to the hypogastrium.

Fourth.—High fever; frequent vomiting; belly swelled and very tender; breathing hurried;* pulse hard and quick; prostration and stupor were depicted in her countenance. Leeches again applied.

Fifth.—Delirium; frequent vomiting. Patient obstinately refused to drink. Mercurial frictions were now employed, two ounces of the ointment being used daily.

Sixth.—Constant delirium; countenance much altered; pulse small and frequent. She soon became comatose, and died; preserving to the last an invincible repugnance to take any fluid.

*Dissection.* In the abdomen a small quantity of sero-purulent fluid. The uterus was imperfectly contracted, and occupied nearly the whole pelvic cavity. The uterine veins were filled with thick yellow pus, which flowed from almost every part of the organ, but more freely from the lateral portions. Towards the fundus uteri, several large lymphatics were seen, also filled with pus: these vessels extended into the substance of the ligamenta lata, and ascended, with the ovarial veins, into the abdomen. The great curvature of the stomach was pierced by three holes, each of about the size of a dollar. The edges of these openings were irregular, fringed, and very soft, and of a very deep brown colour, which extended to a certain distance from the perforations, disappeared insensibly, and again appeared in other parts of the stomach. Some soft adhesions, of recent formation, united these openings to the spleen and diaphragm. The lungs were turgid with blood, and partially covered with circumscribed nodosities, resembling certain hemoptoic engorgements. The brain, heart, liver, and most of the viscera, were soft and flaccid, and presented a singular contrast with the well-developed and richly coloured muscular system.

* We remember a case of puerperal fever, which happened in the early days of our practice, in which the late Dr. GOOCH was consulted. The breathing was rapid, and Dr. G. remarked to us that he never knew a patient recover from the disease when this symptom was present: our subsequent experience corroborates the opinion.—EDITOR.

The morbid condition of the blood, from the entrance of a large quantity of pus into the circulation, did not produce in this patient those general symptoms alone which were observed in the previous cases, but it affected various organs of the body in a decided manner. How, in fact, can the disorganization of the stomach, the circumscribed engorgements of the lungs, and the softened state of all the organs, be explained, unless by some cause, the deleterious influence of which acted upon the whole economy. We may surely conclude, without infringing the limits of rigorous and legitimate induction, that this general cause was the purulent infection that was traced upon examination. Disorganization and perforation of the stomach is often found upon dissection; but the symptoms observed during life, the black colour of the neighbouring parts, the softened state of all the other organs, and, lastly, the adhesions which had begun to form around the perforations, exclude the idea that this was one of the ordinary cases. This softened state of the most dissimilar organs is observed especially in those cases where some deleterious cause exercises its mischievous influence upon the whole system; as in pestilential diseases, typhous fever, the poisoned state of the blood produced by the absorption of miasma, or in animals by the injection of putrid matter into the veins. Daily experience corroborates these opinions. With respect to the circumscribed engorgements of the lungs, as the effect of absorption of matter, M. Tonnellé observes that M. Dance has so ably treated this subject, that it is not necessary for him to dwell particularly upon it. For the same reason, M. T. does not enter upon the consideration of the various purulent collections which are found in the lungs and other parts of the body, after pus has entered the veins.

[To be continued.]

